# Sensory innervation of masseter, temporal and lateral pterygoid muscles in common marmosets

**DOI:** 10.1038/s41598-023-49882-9

**Published:** 2023-12-27

**Authors:** Anahit H. Hovhannisyan, Karen A. Lindquist, Sergei Belugin, Jennifer Mecklenburg, Tarek Ibrahim, Meilinn Tram, Tatiana M. Corey, Adam B. Salmon, Daniel Perez, Shivani Ruparel, Armen N. Akopian

**Affiliations:** 1 Departments of Endodontics, The School of Dentistry, University of Texas Health Science Center at San Antonio, 7703 Floyd Curl Drive, San Antonio, TX 78229-3900 USA; 2 Integrated Biomedical Sciences (IBMS) Program, University of Texas Health Science Center at San Antonio, San Antonio, TX 78229 USA; 3 Departments of Laboratory Animal Resources, University of Texas Health Science Center at San Antonio, San Antonio, TX 78229 USA; 4 Departments of Molecular Medicine, University of Texas Health Science Center at San Antonio, San Antonio, TX 78229 USA; 5 Sam and Ann Barshop Institute for Longevity and Aging Studies, The University of Texas Health Science Center at San Antonio, San Antonio, TX 78229 USA; 6 South Texas Veterans Health Care System, Geriatric Research Education and Clinical Center San Antonio, San Antonio, TX 78229 USA; 7 Oral and Maxillofacial Surgery, University of Texas Health Science Center at San Antonio, San Antonio, TX 78229 USA; 8 Departments of Pharmacology, University of Texas Health Science Center at San Antonio, San Antonio, TX 78229 USA

**Keywords:** Neuroscience, Peripheral nervous system, Sensory processing, Somatosensory system

## Abstract

Myogenous temporomandibular disorders is associated with an increased responsiveness of nerves innervating the masseter (MM), temporal (TM), and lateral pterygoid muscles (LPM). This study aimed to examine sensory nerve types innervating MM, TM and LPM of adult non-human primate—common marmosets. Sensory nerves were localized in specific regions of these muscles. Pgp9.5, marker for all nerves, and NFH, a marker for A-fibers, showed that masticatory muscles were primarily innervated with A-fibers. The proportion of C- to A-fibers was highest in LPM, and lowest in MM. All C-fibers (pgp9.5^+^/NFH^-^) observed in masticatory muscles were peptidergic (CGRP^+^) and lacked mrgprD and CHRNA3, a silent nociceptive marker. TrpV1 was register in 17% of LPM nerves. All fibers in masticatory muscles were labeled with GFAP^+^, a myelin sheath marker. There were substantially more peptidergic A-fibers (CGRP^+^/NFH^+^) in TM and LPM compared to MM. MM, TM and LPM NFH^+^ fibers contained different percentages of trkC^+^ and parvalbumin^+^, but not trkB^+^ fibers. Tyrosine hydroxylase antibodies, which did not label TG, highlighted sympathetic fibers around blood vessels of the masticatory muscles. Overall, masticatory muscle types of marmosets have similarities and differences in innervation patterns.

## Introduction

Myogenous temporomandibular disorders (TMDM) are the most prevalent group of painful orofacial conditions^1–3^. Among musculoskeletal chronic pain conditions, TMDM is the second most widespread after chronic low back pain^4^. Unlike well-localized cutaneous pain, TMDM is often manifested as referred pain to other deep tissues (i.e. eye ache, toothache, headache)^5^. Current knowledge on TMDM related pain mechanisms is limited and further understanding is confounded by conflicting evidence concerning changes in superficial sensitivity seen in patients with craniofacial myalgia^6^. Thus, TMDM is often not accompanied by such clinical signs as histopathologic evidence of injury or inflammation^7^. Therefore, some studies^8^ have classified TMDM as nociplastic pain^9^. Despite these debatable points, there is an agreement that TMDM leads to sensitization of nociceptive and maybe non-nociceptive trigeminal ganglia (TG) sensory neurons innervating the masticatory muscles, leading to increase signal input into the central nervous system^10^.

**Figure 1:**
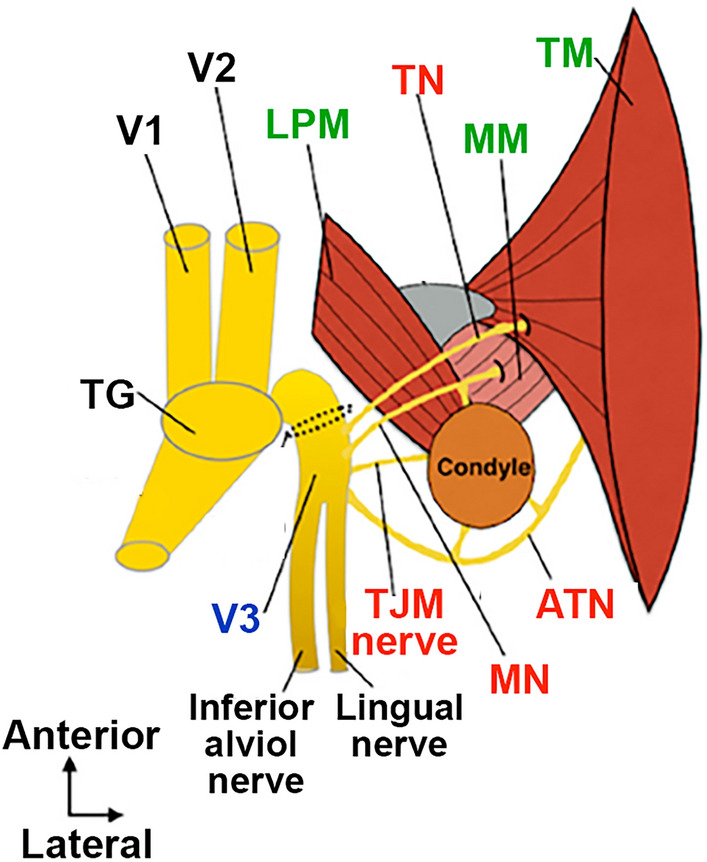
Schematic for sensory neuronal innervation of masticatory muscles. This schematic was adapted from Kucukguven and co-workers publication^12^. V1 – ophthalmic nerve; V2 – maxillary nerve; V3 – mandibular nerve; MN – masseteric nerve; ATN—auriculotemporal nerve; TN – posterior deep temporal nerve; TJM nerve – TJM branch of mandibular nerve; LPM—lateral pterygoid muscle; MM – masseter muscle; TM – temporal muscle.

Increased responsiveness of sensory neurons during TMDM could occur in any muscle types controlling temporomandibular joint (TMJ) articulation, including superficial and in the deep heads of masseter muscle (MM), temporal muscle (TM), medial pterygoid (MPM) closing muscles as well as gliding superior and inferior heads of the lateral pterygoid muscles (LPM)^11,12^ (Fig. 1 adapted from Kucukguven and co-workers^12^). These masticatory muscles are innervated by the masseteric nerve (MN) for MM and LPM, the auriculotemporal (ATN) and TMJ nerves for TMJ and maybe LPM, and temporal nerve (TN) for TM (Fig. 1). ATN plays critical role in the pathophysiology of TMJD^13^, while the MN, posterior deep TN and LPM are sensitized during TMDM^14^ (Fig. 1).

Unlike the inferior alveolar nerve^15,16^, ATN, MN, TN and TMJ nerves are almost uncharacterized and there is only scant information on the types of afferent fibers and their function and plasticity within these branches of the mandibular (V3) nerve. The existing data was generated via extracellular recordings from the submandibular and buccal regions, which are innervated by the ATN. Recordings indicate that ATN may contain C-fibers and slow adapting A-fibers^17–19^. Electrophysiological characterization and immunohistochemistry with sensory nerve fiber markers have shown that mouse MM is predominantly (80–85%) innervated by myelinated fibers^20^. To properly understand and treat TMDM pain further knowledge of the type of peripheral sensory innervation in the masticatory muscles is crucial.

We and others have demonstrated that expression patterns and characteristics of dorsal root (DRG) and TG sensory neurons depends on innervation targets^20–22^. Moreover, there is significant differences in transcriptomic profiles of sensory neurons between rodents and humans^23–25^. These critical differences could explain to some extent why translation of findings in rodents to clinical settings has been challenging, and stresses the need to further investigate sensory systems in other species that might better model the human anatomy and function. In this respect, nonhuman primate (NHP) models are of particular interest for additional pre-clinical testing towards translation. In this study, we used common marmosets (Callithrix jacchus), a well-characterized new world monkey used commonly for research across multiple fields including toxicology, neurological diseases, reproductive biology and aging. We report on immunohistochemistry (IHC) and known molecular markers of sensory nerve types used to characterize and identify the neuroanatomical distributions of these fibers in MM, TM and LPM of naïve adult common marmosets.

## Results

### Localization of sensory nerves in masticatory muscles

**Figure 2:**
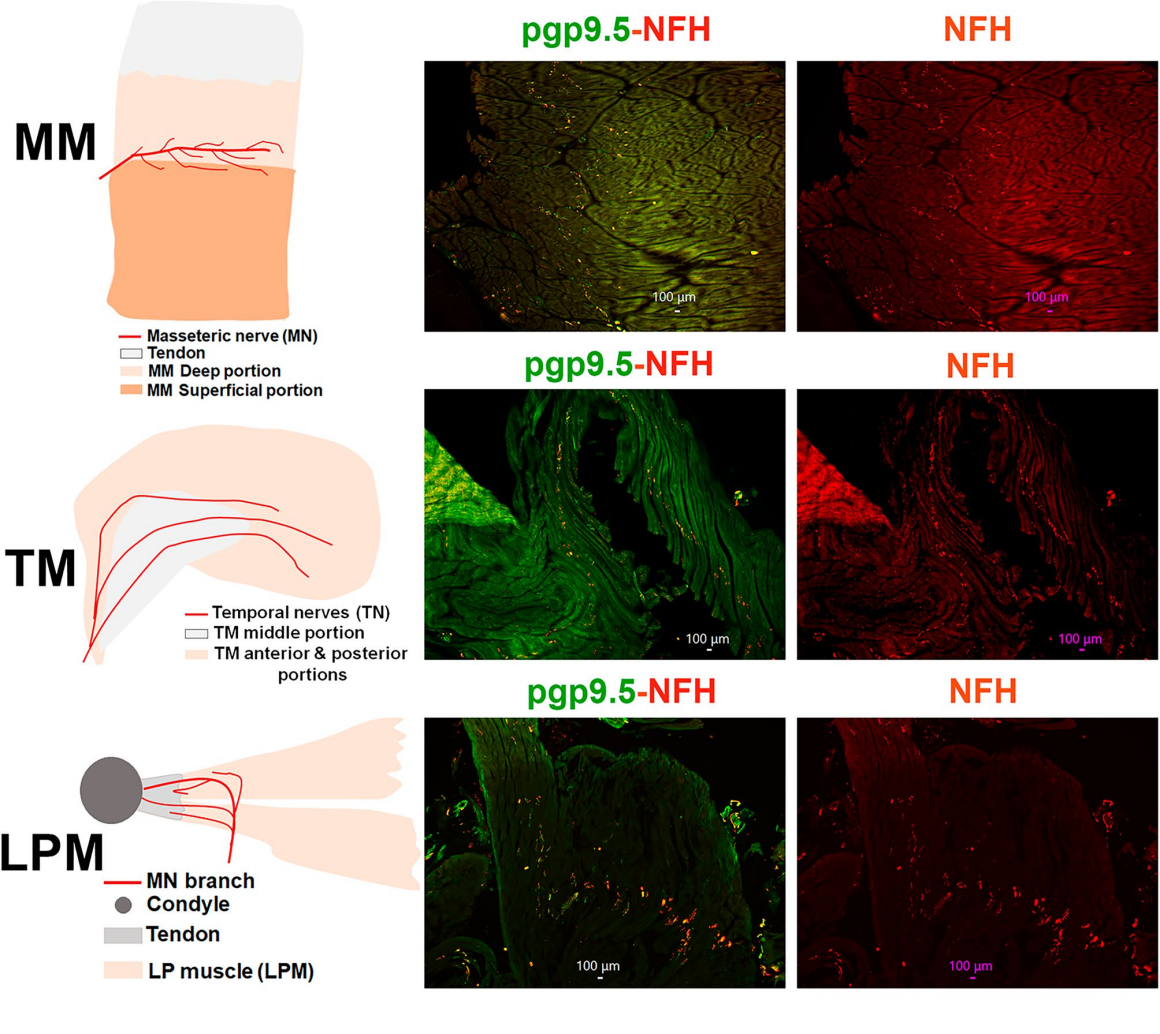
Location of pgp9.5 and NFH-positive fibers in MM, TM and LPM of adult marmosets. *Left column* shows schematic for sensory nerves in marked/specified masticatory muscles. *Middle column* shows expression of pgp9.5 and NFH-positive fibers in MM, TM and LPM. Right column shows expression of NFH-positive fibers in MM, TM and LPM. Pictures from MM, TM and LPM as well as used antibodies and corresponding colors are indicated. Scales are presented in each microphotograph.

Innervation of skin of limbs in animals and humas has even distribution of sensory nerve fibers^26–28^. An innervation pattern for masticatory muscle could be uneven^29^. Thus, mouse MM innervation is localized along route of the MN trunk^20^. Locations of main MN trunk and branches within MM and LPM, and main TN trunk within TM are schematically shown in Fig. 2. We evaluated distribution of all sensory fibers and A-fibers in marmoset masticatory muscles. A-fibers were labeled with NFH^30,31^ and all sensory fibers with pgp9.5^31,32^. Figure 2* upper panels* show that pgp9.5^+^ and NFH^+^ nerves are distributed within a particular area of MM of marmosets. These nerve distribution patterns were similar to those observed in mice^20^, in which nerve ends were located along a line between deep and superficial portions of MM. TM innervation was also highly localized and was detected mainly in an anterior portion of TM (Fig. 2* middle panels*). Accordingly, TM was heavily innervated in TM portion connected to the tendon, which is extended to mandible. LPM were innervated along MN trunk traveling toward condyle/TMJ (Fig. 2* bottom panels*). Overall, masticatory muscles of marmosets had localized sensory nerve innervation along main trunks of MN and TN.

### Detections and quantifications of sensory nerves in masticatory muscles of marmosets

There are several approaches to detect and quantify fibers. These approaches have stronger and weaker points, and applied depending on aims and tool availability. Thus, reporter lines cannot be applied for marmosets as they are used in mice. Hence, sensory fibers are detected using cryosections and antibodies, which often have weak expression levels in neurites. Moreover, NHP tissues were often not optimal due to more complicated dissection procedures comparing to those in mice. To mitigate these drawbacks, first, we counted clear identified nerve fibers with length of at least 20 µµ. This measure allowed to avoid counting possible labeling of non-neuronal cells and/or artifacts in muscles. Second, we used validated antibodies with minimal-to-no autofluorescence and those producing expression patterns in marmoset trigeminal ganglion (TG) neurons. Third, antibodies used in these studies were validated this way using TG labeling and auto-fluorescent validation of secondary antibodies as described before^20,33,34^.

Image J provides several approaches to quantify nerve fiber length^35,36^. This approach is useful for studies on neuronal morphology and neurite outgrowth. The main drawback of this approach is that the length of individual fibers can vary with each section due to unequal distribution of fibers throughout the depth of the tissue. Measuring length of fibers is not an appropriate approach for our study, since it is almost exclusively used to quantify nerve degeneration or regeneration processes, which are outside of scope of this study. Another approach assesses density of fibers using Image J platform^37,38^. This approach is required even distribution of fibers through tissues, which is not the case for masticatory muscle (Fig. 2). Moreover, measurement of density will include all labeled structures by antibodies. Therefore, non-neuronal cell labeling and non-specific labeling will contribute density data and compromise outcome.

For this study, we use the manual quantification of fiber numbers, which is extensively used to assess neuropathies in animal models^39^ and clinic^40^. This approach selection were made for several reasons: (a) uneven distribution of fibers in masticatory muscles; (b) this study does not aim investigating regeneration or degeneration; (c) it is used for neuropathy assessments, since provided for greater control over the process especially when identifying nerve fibers from artifact or debris and (d) for this particular study, quantification of fiber density and length does not provide extra information on type of fibers innervating masticatory muscles.

### Distribution of C- and A-fiber markers in the masticatory muscles

Original classifications of A and C fibers as well as subclasses Aδ, Aβ and Aα were made according to conduction velocity and electron microscopy-based identification of myelin sheath parameters^41,42^. Extensive multi-year research associated C- and A-fiber subclasses with specific markers, which widely used to link neurites or neurons to C-, A-fiber or its sub-types ^43–46^. We will use these markers to refer to neurites as C-, A-, Aδ-, Aβ- or Aα-fibers.

To examine the proportion of C-fibers compared to A-fibers in male marmoset masticatory muscles, we labeled A-fibers with NFH^30,31^. All sensory fibers (C- and A-fibers) were identified by labeling with pgp9.5, which was used in numerous studies for identification of sensory neurites^31,32^ and distinguished sensory neurite from motor axons^47^. However, there are reports showing pgp9.5 labeling in motor axons in muscles^48^. Pgp9.5^+^ and NFH^+^ fibers were detected at the junction of superficial and deep heads of MM (Fig. 2). Both Pgp9.5^+^ and NFH^+^ fibers had localized distribution in TM and LPM as well (Fig. 2). Moreover, superior and inferior LPM had similar distribution patterns for Pgp9.5^+^ and NFH^+^ fibers (Fig. 2).

**Figure 3:**
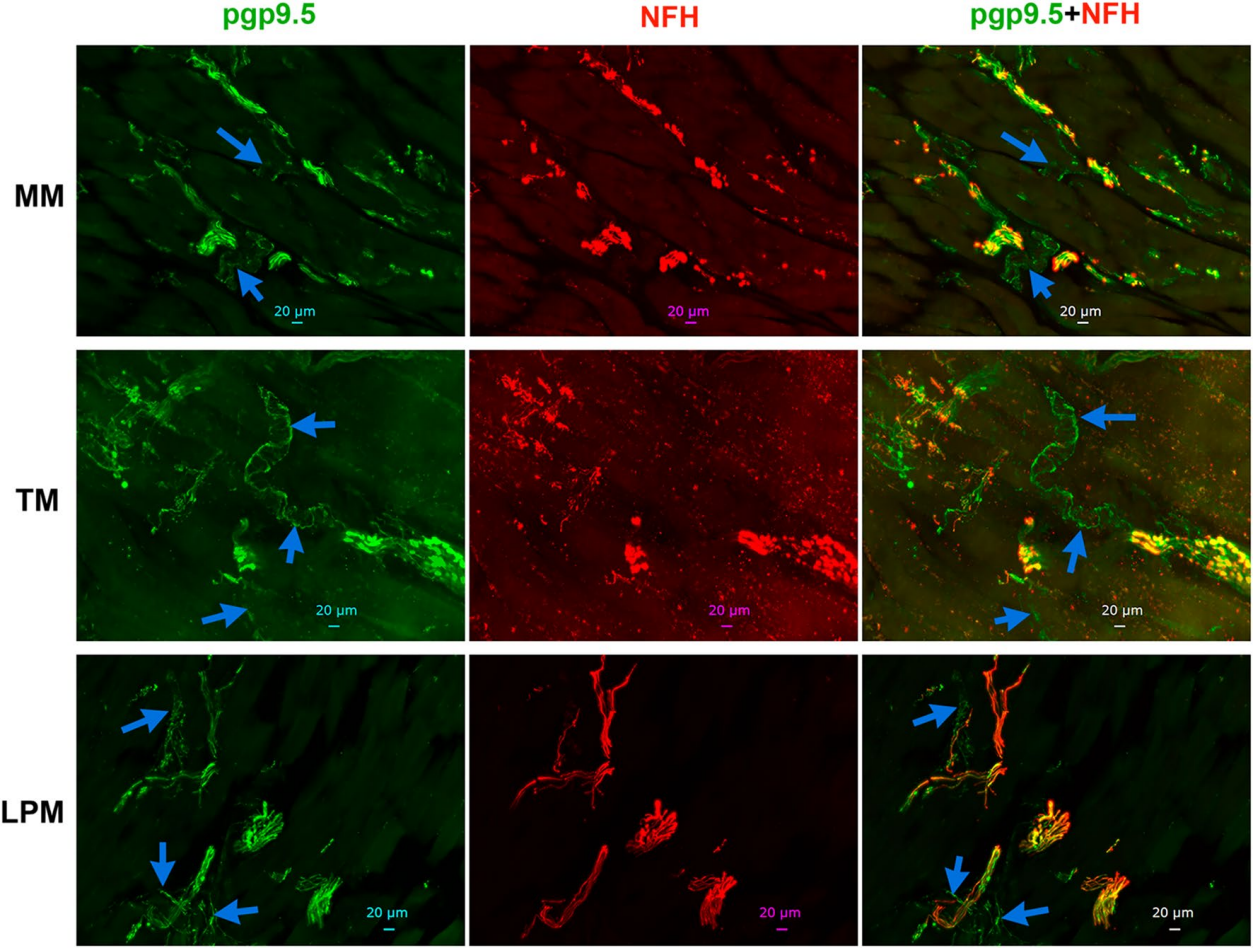
Representation of pgp9.5 and NFH-positive fibers in MM, TM and LPM of adult marmosets. Representative micro-photographs show relative expression of NFH (A-fibers) and pgp9.5 (all fibers) positive fibers in MM, TM, and LPM of adult male marmosets. Blue arrows indicate pgp9.5^+^/NFH^-^ fibers. Pictures from MM, TM and LPM, as well as antibodies used, and corresponding colors are indicated. Scales are presented in each microphotograph.

**Figure 4:**
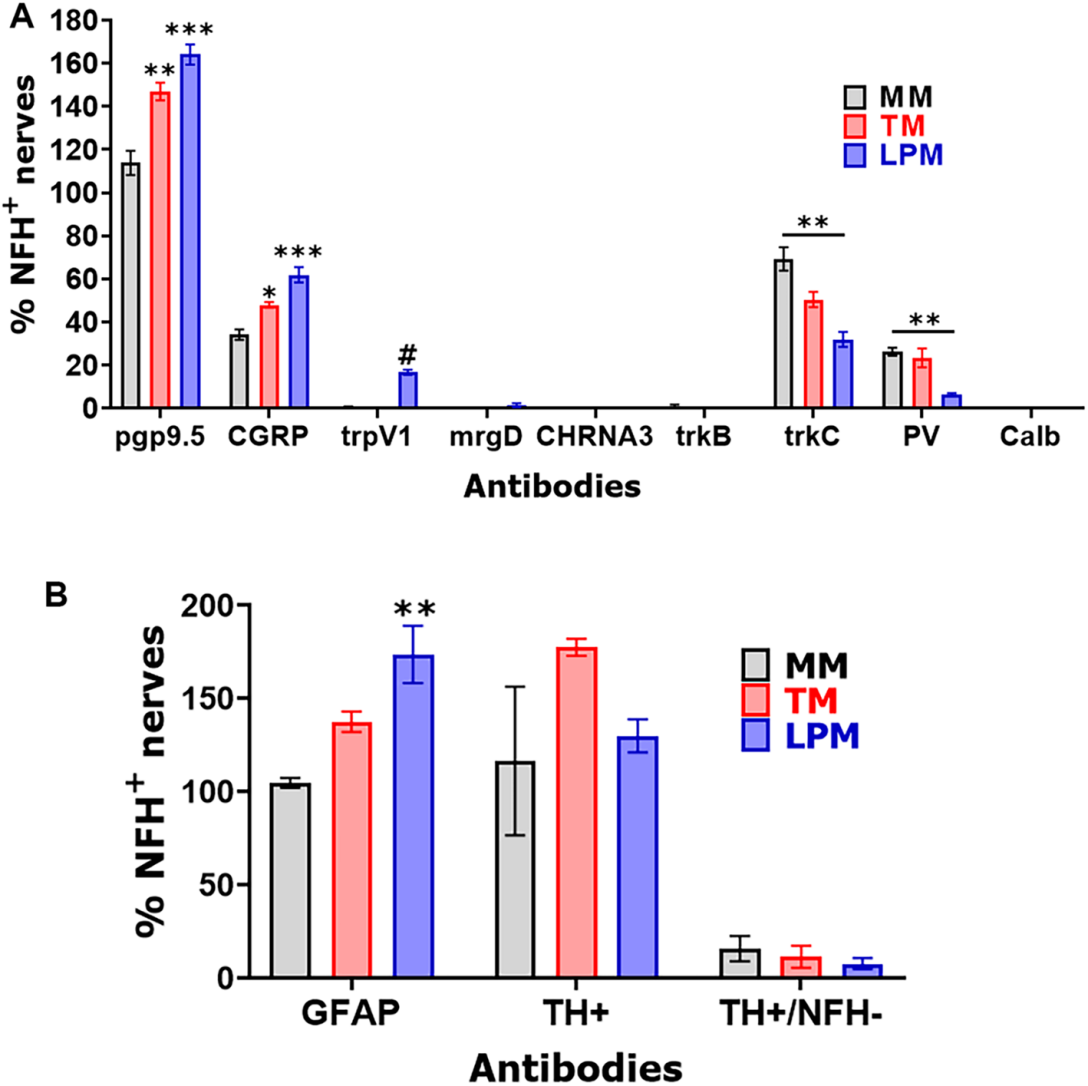
Percentages of different fiber types in MM, TM and LPM of adult marmosets. (**A**) Baragraphs reflect percentages of marker-positive sensory fibers relative to NFH (A-fibers) in MM, TM, and LPM of adult male marmosets. (**B**) Baragraphs reflect percentages of GFAP^+^ (myelinated) and TH^+^ (sympathetic) fibers relative to NFH (A-fibers) in MM, TM, and LPM of adult male marmosets. X-axis denotes markers and tissue types (i.e. MM, TM and LPM). N = 3 for MM and N = 2 for TM and LPM.

We measured proportion of different fibers relatively to NFH, since unlike NFH antibodies produced in chickens, a majority of antibodies, including pgp9.5, were produced in rabbits. Alternative pgp9.5 monoclonal antibodies generated in mice and reported previously^37,38^ showed strong non-specific secondary antibody-independent labeling and high auto-fluorescence. NFH and pgp9.5 labeling overlapped in a majority of sensory nerves in MM (Fig. 3; Suppl Fig. 1). TM and LPM had substantially more proportion of C-fibers (pgp9.5^+^/NFH^-^) compared to MM (Figs. 3 and 4A, Suppl Fig. 1; 1-way ANOVA; F (2, 7) = 26.76; *P* = 0.0005; n = 3–4). Thus, we estimated that MM had ≈15% of C-fibers and ≈85% of A-fibers, while TM and LPM had ≈35–45% C-fibers and ≈55–65% A-fibers (Fig. 4A). Overall, masticatory muscles were found to be primarily innervated by A-fibers; and MM contained the highest proportion of A-fibers, whereas LPM had lowest.

**Figure 5:**
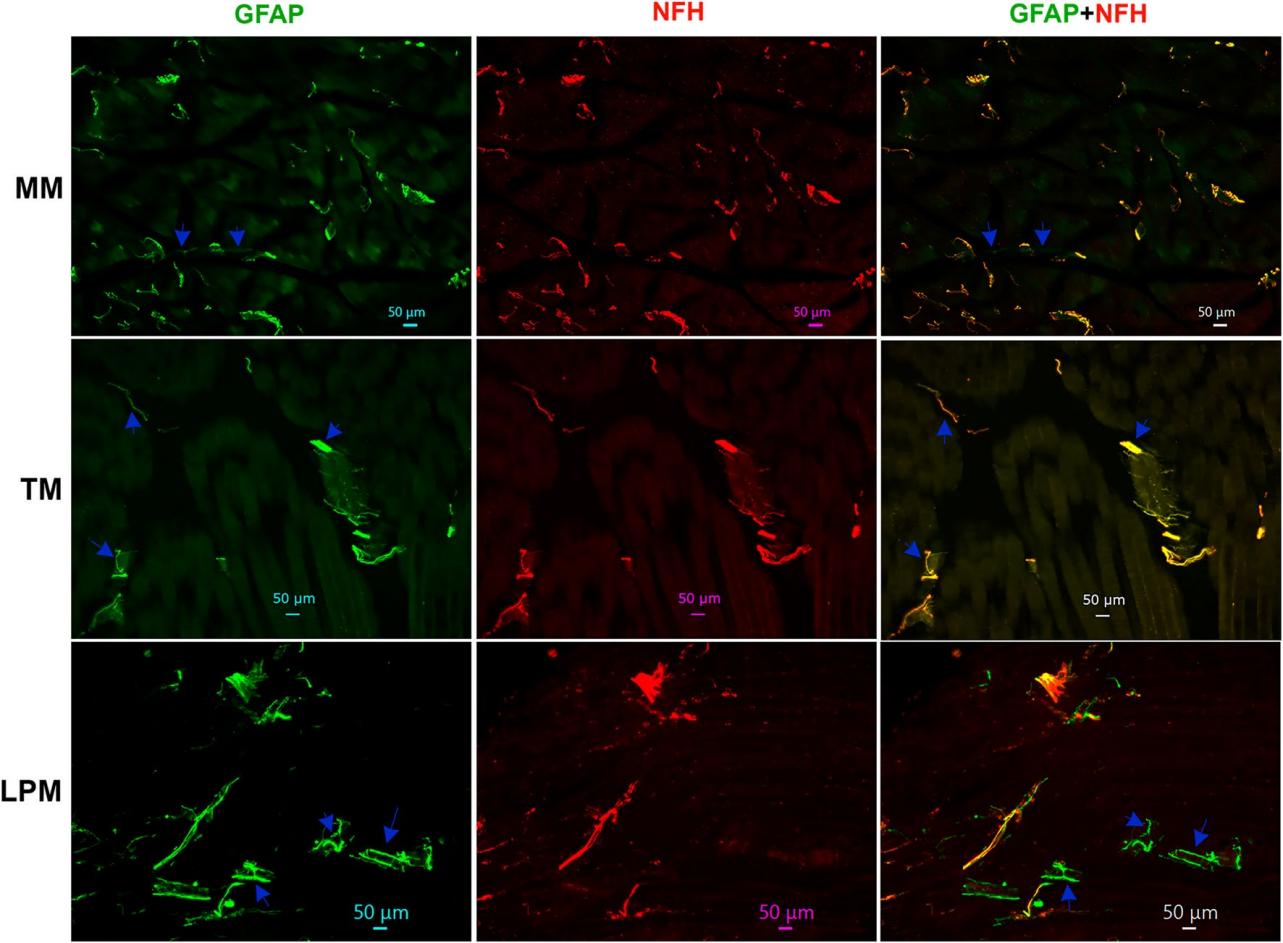
*Representations of myelinated fibers in MM, TM and LPM of adult marmosets*. Representative micro-photographs show GFAP myelinated fiber distributions relative to NFH^+^ fibers in MM, TM, and LPM of adult male marmosets. Blue arrows indicate GFAP^+^/NFH^-^ fibers. Pictures from MM, TM and LPM as well as used antibodies and corresponding colors are indicated. Scales are presented in each microphotograph.

A-fibers are traditionally considered myelinated, while C-fibers are unmyelinated^31^. Nevertheless, it was reported that in certain tissues several unmyelinated C-fibers could be wrapped together by myelin sheath^34,49^. To examine whether several C-fibers are wrapped by myelin sheath in masticatory muscles of adult marmosets, we labeled myelin with GFAP antibodies. A majority of reports, except few^50^, consider GFAP as a marker for astrocytes, Schwann cells and satellite glial cells. We additionally validated GFAP antibodies in marmosets trigeminal ganglia (TG) (Suppl Fig. 2)^51^. All NFH^+^ nerves in MM were labeled with GFAP, but about 4% nerves were GFAP^+^/NFH^-^ (Figs. 4B, 5). NFH^+^ nerves in TM and LPM were also co-labeled with GFAP (Fig. 5). Proportions of GFAP^+^/NFH^-^ fibers in TM and, especially LPM were significantly higher compared to MM (Figs. 4B, 4; 1-way ANOVA; F (2, 4) = 20.03; *P* = 0.008; n = 3). We found that TM and LPM contained ≈28% and ≈43% GFAP^+^/NFH^-^ fibers, respectively (Fig. 4B). Altogether, our data point to possibility that several un-myelinated C-fibers are organized into fiber bundles by wrapping them with GFAP^+^ myelin sheath. This type of C-fiber organization was previously reported for dura mater^52^. This wrapping with myelin sheath does not make C-fibers as a subset of A-fibers, since for A-fibers, every neurite is individually wrapped in myelin sheath.

### Distributions of CGRP^+^ peptidergic nerves in the masticatory muscles

**Figure 6:**
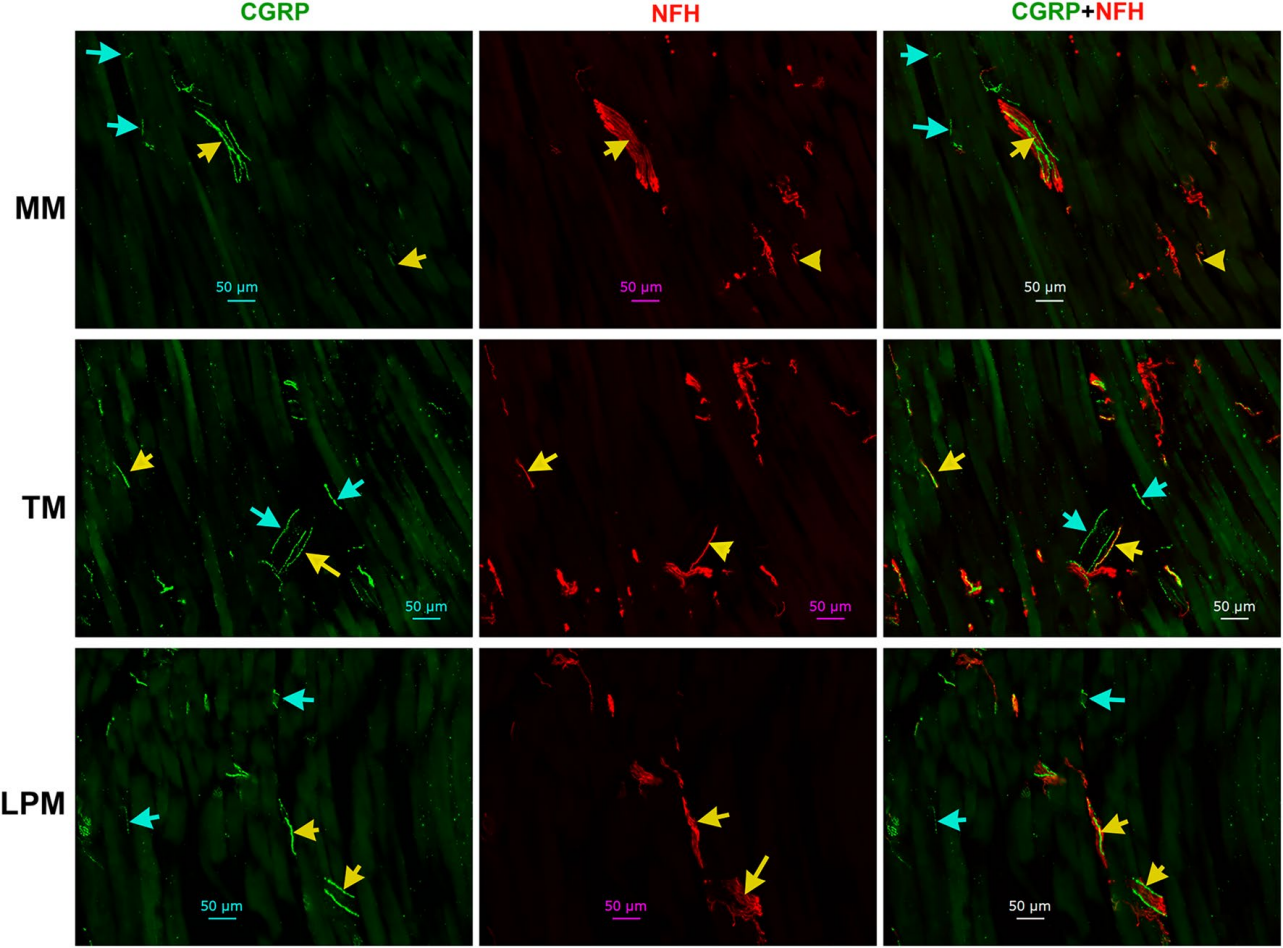
Distribution of peptidergic fibers in MM, TM and LPM of adult marmosets. Representative micro-photographs show CGRP-positive peptidergic fiber distributions relative to NFH^+^ fibers in MM, TM, and LPM of adult marmosets. Yellow arrows indicate CGRP^+^/NFH^+^ fibers and cyan arrows show CGRP^+^/NFH^-^ fibers. Pictures from MM, TM and LPM as well as used antibodies and corresponding colors are indicated. Scales are presented in each microphotograph.

A standard marker for peptidergic nerves and neurons is CGRP^43,53^. We used validated anti-CGRP antibodies, which exhibited strong labeling in a subset of TG neurons in adult male marmosets (Suppl Fig. 2). Compared to CGRP^+^ signal strength in marmosets TG, CGRP^+^ labeling in masticatory muscle was relatively dimmer (Fig. 6; Suppl Figs. 1, 3). MM had about 34% of CGRP^+^ nerves, and only ≈10% were CGRP^+^/NFH^-^ (Figs. 4A, 6; Suppl Fig. 3). The proportion of CGRP^+^ fibers were higher in TM and particularly in LPM compared to MM (1-way ANOVA; F (2, 7) = 29.34; *P* = 0.0004; n = 3–4; Figs. 4A, 6; Suppl Fig. 3). Nevertheless, less than half of CGRP^+^ positive fibers were GFAP^+^/NFH^-^ in TM and LPM (Figs. 4A, 6; Suppl Fig. 3). These data indicate that peptidergic nerves in the masticatory muscles, especially MM, are predominantly composed of A-fibers.

### Distributions of TrpV1 and MrgprD-positive nerves in the masticatory muscles

**Figure 7:**
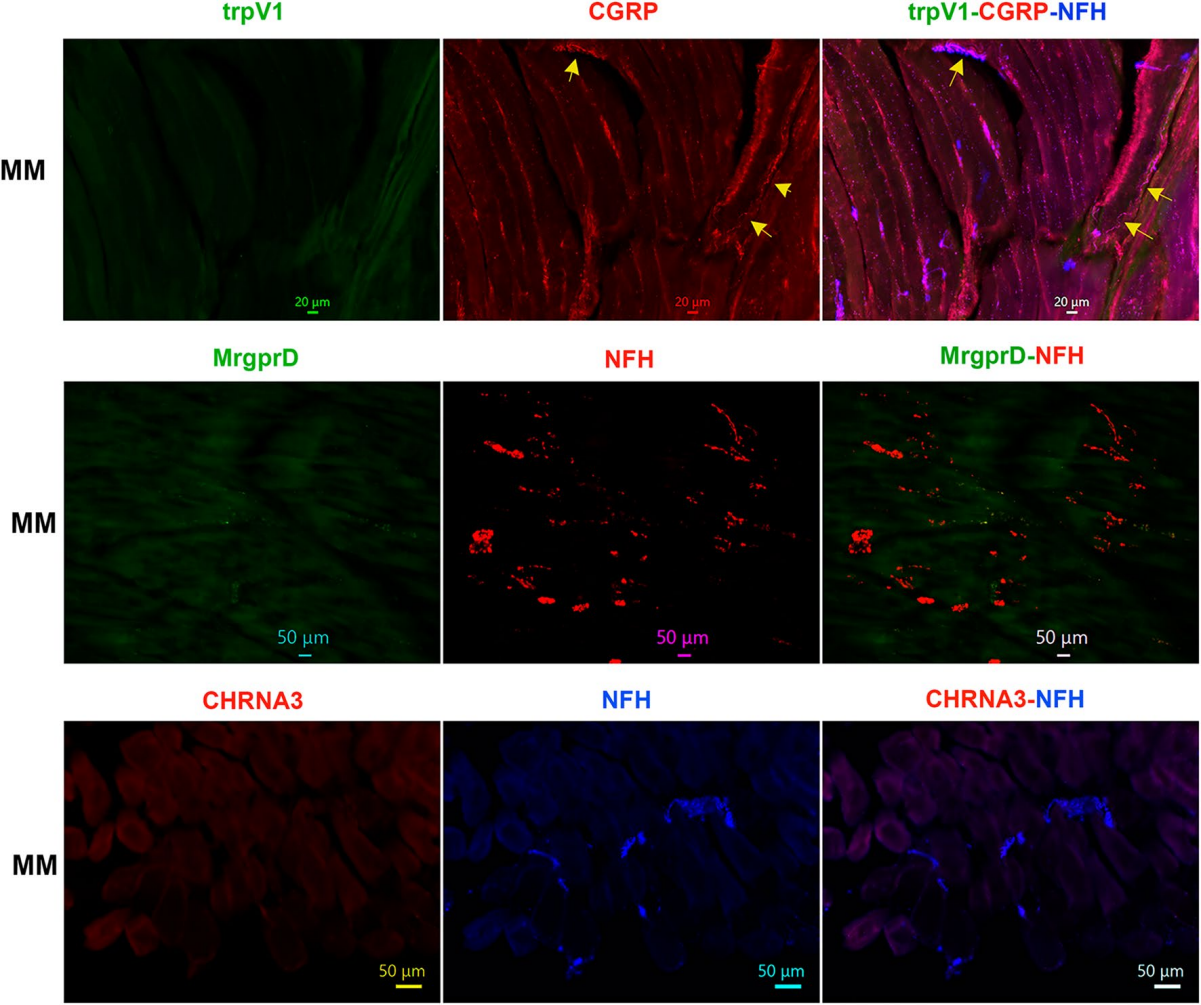
Distribution of trpV1-, MrgprD and CHRNA3-positive fibers in MM of adult male marmosets. Representative micro-photographs show trpV1^+^, mrgprD^+^ non-peptidergic fiber, and CHRNA3^+^, marker for “silent” nociceptors, fiber distributions relative to CGRP^+^ and/or NFH^+^ nerves in MM of adult male marmosets. Yellow arrows in top row panels mark some CGRP^+^/NFH^+^/trpV1^-^ fibers. Pictures from MM as well as used antibodies and corresponding colors are indicated. Scales are presented in each microphotograph.

**Figure 8:**
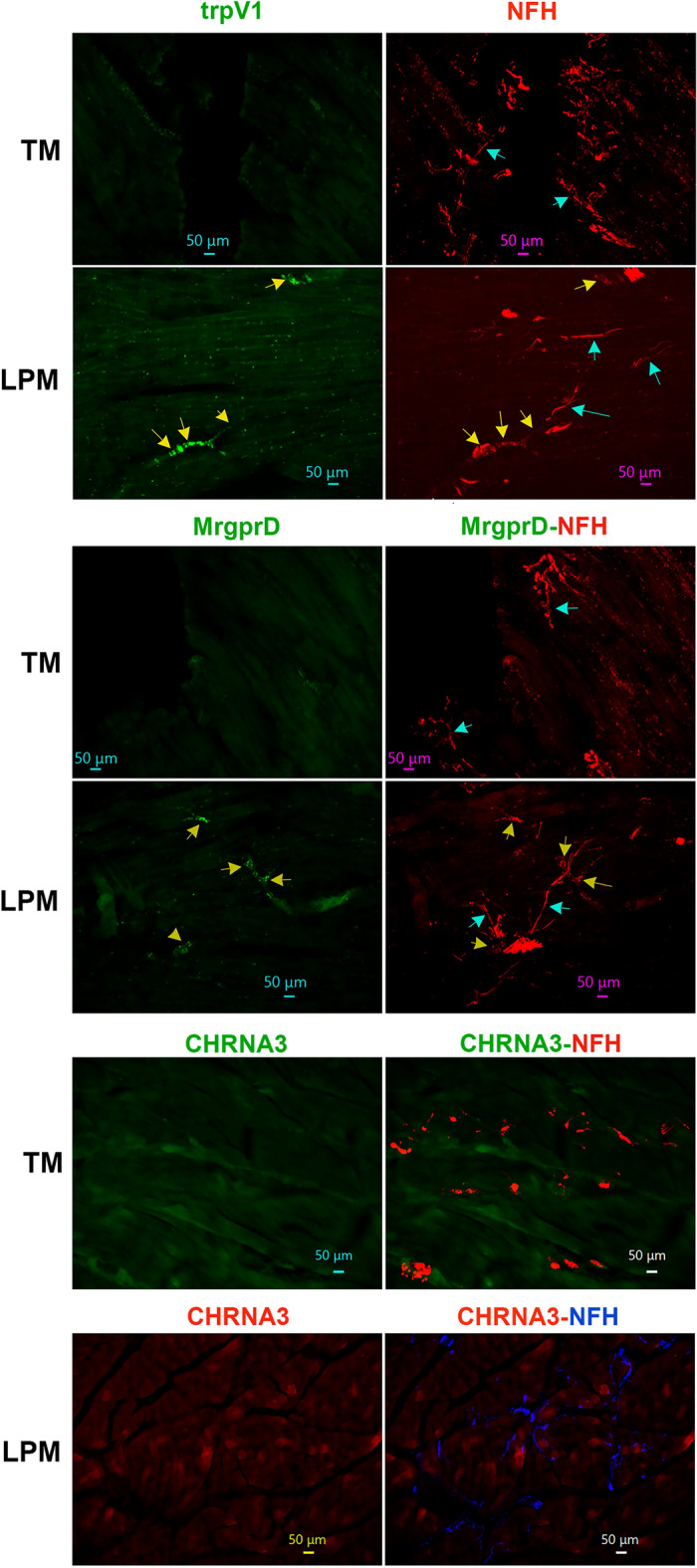
Distribution of trpV1-, MrgprD and CHRNA3-positive fibers in TM and LPM of adult marmosets. Representative micro-photographs show trpV1^+^, mrgprD^+^ and CHRNA3^+^ fiber distributions relative to NFH^+^ nerves in TM and LPM of adult male marmosets. Yellow arrows (panels for trpV1 and mrgprD) indicate trpV1^+^/NFH^+^ or mrgpr^+^/NFH^+^ fibers, while cyan arrows (panels for trpV1 and mrgprD) show trpV1^-^/NFH^+^ or mrgpr^-^/NFH^+^. Pictures from TM and LPM as well as used antibodies and corresponding colors are indicated. Scales are presented in each microphotograph.

Data from Figs. 1, 2, 3, 4, 5, 6 imply that masticatory muscles have relatively smaller subset of C-fibers compared to skin and dura mater and are predominantly innervated by A-fibers^52,54,55^. Here, we have evaluated expressions of trpV1, a marker for a subset of C-fiber sensory neurons^43^, and MrgprD, a marker for non-peptidergic C-fiber sensory neurons^43,53^, in the masticatory muscles. TrpV1 and MrgprD antibodies were validated on TG sections, and produced strong labeling in subsets of adult male marmoset TG neurons (Suppl Fig. 1). As expected from pgp9.5, NFH and CGRP co-labeling, MrgprD fibers were not detected in MM, TM and LPM (Figs. 4A, 7, 8). Surprisingly, despite capsaicin produced behavioral responses in animals and humans, trpV1^+^ fibers were not identified in MM, TM and LPM (see “Discussion”; Figs. 4A, 7, 8). Nevertheless, LPM sections generated from one of 4 animals contained some nerves bundles showing both trpV1 and MrgprD labeling as well as NFH^+^ fibers (Figs. 4A, 8). This unusual labeling for trpV1 and MrgprD in these LPM sections could belong to stretches of MN or ATN nerve trunks, which pass through LPM towards the TMJ ligament (see “Discussion”; Fig. 2 schematic). Overall, we found that MM, TM and LPM did not show immunoreactivity for trpV1 or MrgprD, a marker for non-peptidergic sensory fibers.

### Expression of a marker for “silent” nociceptors in the masticatory muscle nerves

Certain nociceptors were labeled “silent” nociceptors, since these neurons are insensitive to piezo device stimulation or respond only to very high-threshold in vitro or in vivo mechanical stimuli. These nociceptors are a subset of C-fibers in DRG and identified by expression of the nicotinic acetylcholine receptor subunit alpha-3 (CHRNA3)^56^. We used validated anti-CHRNA3 antibodies, which show labeling of fibers in the tongue of common marmosets (Suppl Fig. 1), to identify CHRNA3^+^ fibers in masticatory muscles. Unlike NPH tongue^34^, MM, TM and LPM did not express CHRNA3 nerves at a detectable level (Figs. 7, 8). We also attempted to label marmoset masticatory muscles with several different human PIEZO2 antibodies. These antibodies were not suitable in labeling in marmosets, since they have either did not show any signal or labeling could have been attributed to autofluorescence.

### Expressions of A-fiber markers in the masticatory muscle nerves

**Figure 9:**
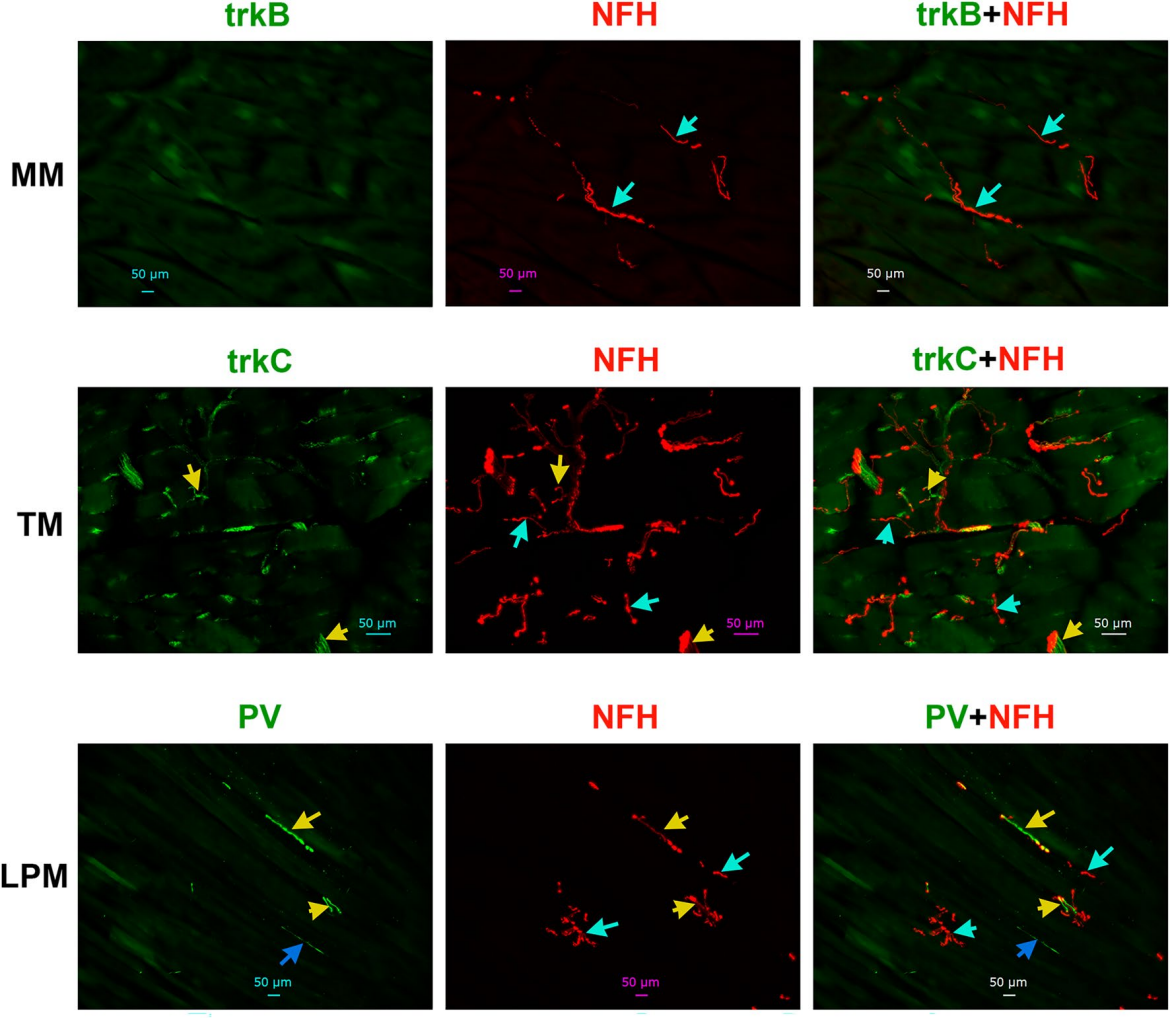
Distribution of trkB, trkC and parvalbumin (PV)-positive fibers in MM of adult marmosets. Representative micro-photographs show expression of markers for non-nociceptive neurons, trkB, trkC and parvalbumin (PV), in sensory fibers in MM of adult marmosets. NFH was used to outline all A-fibers in the tissues. Top panel: Cyan arrows indicate trkB^-^/NFH^+^ fibers. Middle panel: Yellow arrows indicate trkC^+^/NFH^+^ fibers, while cyan arrows show trkC^-^/NFH^+^. Bottom panel: Yellow arrows indicate PV^+^/NFH^+^ fibers, while cyan arrows show PV^-^/NFH^+^ fibers and blue arrow shows PV^+^/NFH^-^ fiber. Pictures from MM as well as used antibodies and corresponding colors are indicated. Scales are presented in each microphotograph.

**Figure 10:**
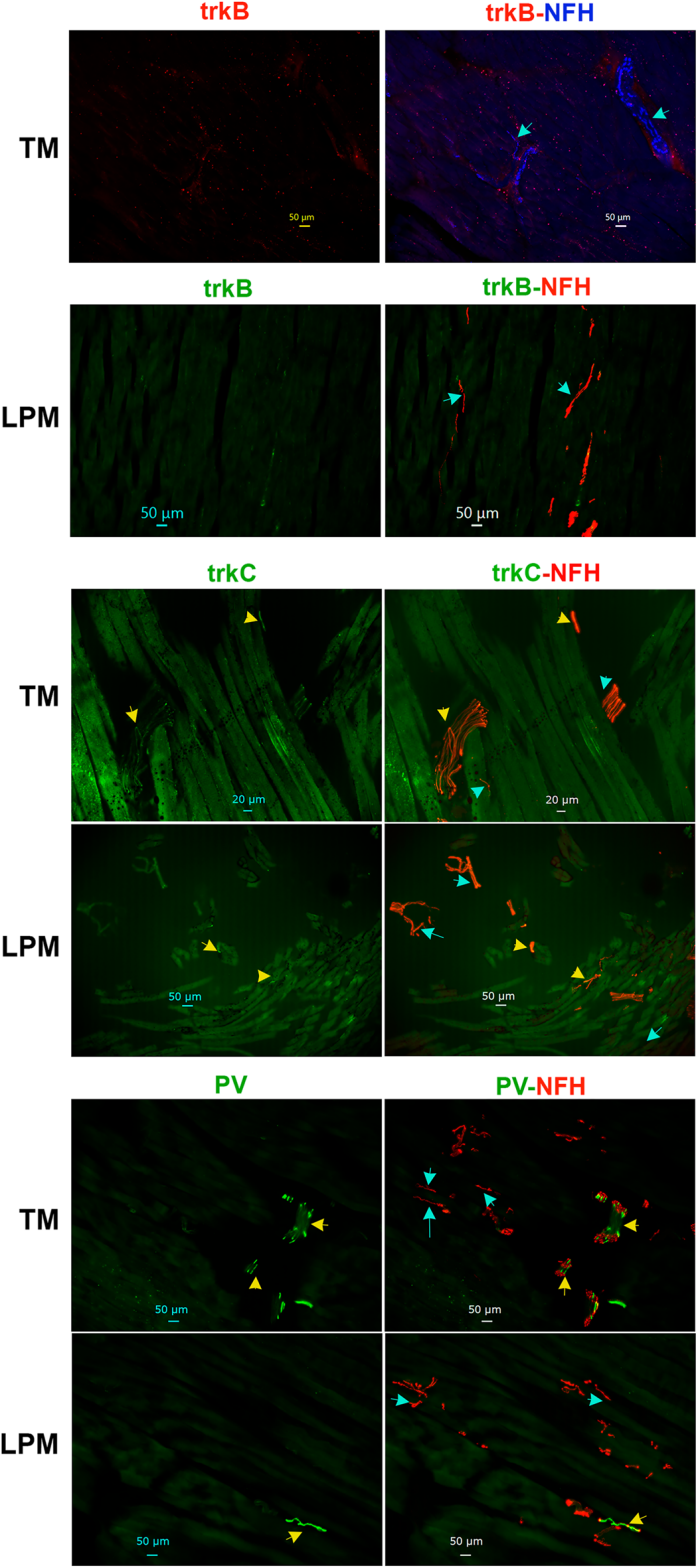
Distribution of trkB, trkC and parvalbumin (PV)-positive fibers in TM and LPM of adult marmosets. Representative micro-photographs show expression of trkB, trkC and PV sensory fibers in TM and LPM of adult marmosets. NFH was used to outline all A-fibers in the tissues. Top panels: Cyan arrows show trkB^-^/NFH^+^. Middle panel: Yellow arrows show trkC^+/^NFH^+^, while cyan arrows mark trkC^-^/NFH^+^ sensory fibers. Bottom panels: Yellow arrows show PV^+/^NFH^+^ , while cyan arrows mark PV^-^/NFH^+^ sensory fibers. Pictures from TM and LPM as well as used antibodies and corresponding colors are indicated. Scales are presented in each microphotograph.

Single-cell RNA sequencing data and electrophysiology studies on reporter mice indicate that tyrosine hydroxylase (TH), trkB, trkC, parvalbumin (PV) and calbindin-28d (Calb) are markers for DRG low-threshold mechanoreceptor (LTMR) sensory neurons and cutaneous A-fibers^43,46,53,57^. Validated antibodies for trkB, trkC, PV and Calb labeled a subset of TG neurons in adult male marmosets (Suppl Fig. 4), whereas we observed no TH labeling of neurons or non-neuronal cells in marmosets TG (Suppl Fig. 1). TrkB as a marker for Aδ-LTMR in skin produced in some MM sections very weak labeling, which was resembled to auto fluorescence or leak from adjusted fluorescent channels (NFH). We have interpreted this data as absence of trkB fibers in MM (Figs. 4A, 9)^58^. No TM and LPM sections showed detectable trkB labeling (Figs. 4A, 10). A marker for Aβ-LTMR, trkC, was present more than 70% of NFH^+^ fibers in MM (Figs. 4A, 9)^43,57^. In contrast, trkC was identified in ≈40–50% of A-fibers in TM and LPM (Figs. 4A, 10 and Suppl Fig. 5). The percentages of trkC^+^ A-fibers in TM and LPM were lesser than those found in the MM (1-way ANOVA; F (2, 7) = 16.74; *P* = 0.0022; n = 3–4; Figs. 4A, 9, 10). Certain trkC^-^/NFH^+^ and trkC^+^/NFH^+^ fibers were located at the junction of muscle and tendon in TM (Suppl Fig. 5). PV is another traditional marker for certain Aβ-LTMR and proprioceptors in DRG^43,57^. Relatively robust labeling with PV antibodies was detected among NFH^+^ nerves in MM and TM, and to a lesser extent in LPM (1-way ANOVA; F (2, 7) = 16.20; *P* = 0.0024; n = 3–4; Figs. 4A, 9, 10). Calb is a marker for the Aβ-field (aka NF2) sensory neuron type^43,44,59^. Calb^+^ nerves were not found in MM, TM or LPM (Fig. 4A). Overall, our data suggest that the masticatory muscles are innervated by A-fibers, including A-LTMR, which are defined by expression patterns of trkC and PV. Data suggest that types of A-fibers are distinct for MM, TM and LPM. MM had the highest proportion of A-LTMR among NFH^+^ nerves (i.e. A-fibers).

### Innervation of blood vessels in the masticatory muscles

**Figure 11:**
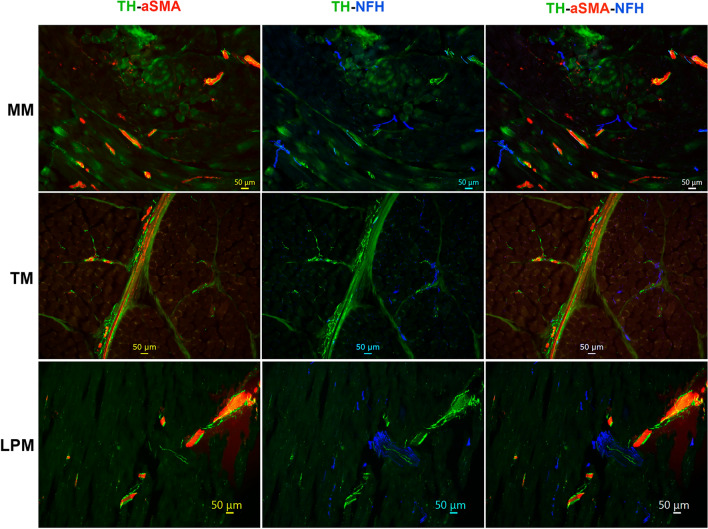
Location of sympathetic fibers in MM, TM and LPM of adult marmosets. Representative micro-photographs show the location of tyrosine hydroxylase (TH)-positive sympathetic fibers relative to blood vessels outlined with smooth muscle marker alpha-smooth muscle actin (α-SMA) and sensory A-fibers labeled with NFH in MM, TM, and LPM of adult male marmosets. Pictures from MM, TM and LPM as well as used antibodies and corresponding colors are indicated. Scales are presented in each microphotograph.

Muscle ischemia could play an important role in initiation of TMDM. Hence, we investigated innervation of muscle blood vessels. We have used TH as a marker for sympathetic nerves. TH is also a marker for cutaneous C-LTMR^57,60^. However, we could not identify TH^+^ sensory neurons in marmosets TG (Suppl Fig. 2). Expansive presence of TH^+^ nerves was revealed in the masticatory muscles (Figs. 4B, 11). The numbers of TH^+^ fibers were comparable to NFH^+^ ones in all studied masticatory muscles (Figs. 4B, 11; 1-way ANOVA; F (2, 4) = 0.95; *P* = 0.46; n = 3). A majority of TH^+^ fibers were not co-localized with NFH, but positioned around alpha-smooth muscle actin (α-SMA^+^) blood vessels in MM, TM and LPM (Figs. 4A, 11; 1-way ANOVA; F (2, 4) = 0.45; *P* = 0.67; n = 3). Overall, TH^+^ nerves representing sympathetic fibers are located around blood vessels in MM, TM and LPM. Certain portion of sensory NFH^+^ fibers are also in a vicinity of masticatory muscle blood vessels as well as nearby of TH^+^ fibers (Fig. 11).

## Discussion

The etiology and pathogenesis of TMDM is poorly understood^6,61,62^. However, there is an agreement that TMD-M leads to sensitization of nociceptive and non-nociceptive TG sensory neurons innervating the masticatory muscles, and thereby enhancing signal input into the central nervous system and inducing pain^10^. Thus, in order to improve our knowledge about chronic TMDM and possible therapeutic targets, it is essential to characterize sensory nerves in the masticatory muscles. Such information will have higher translatability to clinical application when it is obtained from either humans or animal species that have similar neuroanatomy to humans. Current single-cell RNA sequencing data imply that DRG and TG sensory neurons have similar neuronal clusters^25,59,63^. Nevertheless, it was demonstrated for several tissues that expression patterns for a variety of genes and electrical activity of DRG and TG sensory neurons depends on innervation targets^20–22^. TMDM is prevalent in females (70%) compared to males (30%). Studies on gene expressions in females and males sensory neurons showed that some genes show differential and sex-depndent expression^64–66^. However, sensory neuron types are the same in males and females^24,43,44^. Taken together, this study did not evaluate sex-dependent expressions of fibers and was conducted on adult (3–11 years-old) common marmosets, a nonhuman primate species that more closely represents human physiology, genetics, and anatomy than do rodent models. We selected key masticatory muscles, MM, TM and LPM, for this study, since they innervation could be distinct.

**Figure 12:**
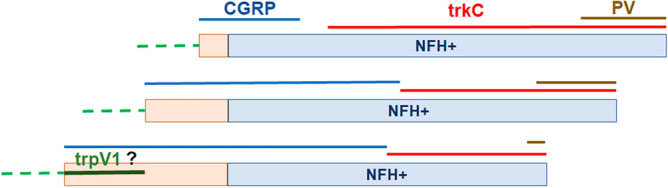
Schematic for sensory neuron subtypes and marker expression in masticatory muscles of adult marmosets. Masticatory muscles type (i.e. MM, TM and LPM) are indicated from left. Sensory nerve groups innervating MM, TM or LPM are marked by lines and corresponding marker. ? indicates unclear presence of trpV1 marker in LPM. Green dush lines marker absence of MrgprD non-peptidergic nerves.

Masticatory muscles, especially MM, contained a substantially higher proportion of A relatively to C-fibers (Fig. 12). This innervation pattern is similar to those observed in mouse MM, which is predominantly innervated by A-fibers^20^. C-fibers in the masticatory muscles of adult male marmosets are peptidergic, lack non-peptidergic neuronal marker mrgprD and not labeled with trpV1 antibodies. However, some sections showed trpV1^+^ and MrgprD^+^ fibers in LPM. Thus, we observed possible mrgprD and trpV1 immunoreactivity in stretches of NFH containing nerve trunks in LPM, while mrgprD^+^/NFH^-^ and trpV1^+^/NFH^-^ nerves were absent in LPM. Nevertheless, such labeling was not consistent and was not observed in all marmosets. This phenomenon could be explained by the fact that LPM is innervated by a mandibular nerve branch—MM and ATN, which travels through LPM on the way to innervating TMJ ligaments^12^. Therefore, trpV1^+^ and mrgprD^+^ fibers could belong to such a nerve trunk targeting TMJ. Besides, it could not be excluded that this labeling in some marmoset tissues could be non-specific. Absence of mrgprD^+^ and trpV1^+^ immunoreactivities and lack of trpV1-GFP^+^ and mrgprD-TdTom^+^ nerves were also reported in mouse MM^20^. It is surprising that we could not detect trpV1^+^ nerves in primates as well as mouse masticatory muscles^20^. Behavioral experiments on rodents and clinical data demonstrated that intramuscular capsaicin (> 10 µγ) elicits nociception in rodents and pain and crump-like sensation for humans^55,67–70^. One of possible explanations for this difference is that low levels of trpV1 on nerves is sufficient for responses to capsaicin stimulation. In contrast, higher expression of trpV1 on fibers is required to reveal immunoreactivity. Another possibility is that trpV1 is expressed on non-neuronal cells surrounding sensory fibers; and capsaicin sensitize nerves indirectly by activating surrounding non-neuronal cells. It is also possible that injected capsaicin defused to a mucosal part, loose areolar and subcutaneous as well as pericranium tissues of facial skin covering muscles, which contain trpV1^+^ nerves. Besides, mrgprD and trpV1 immunoreactivity, masticatory muscles also lack the marker for the “silent” nociceptor, CHRNA3^56^.

C-fibers are traditionally considered un-myelinated fibers. However, it was reported for dura mater that several C-fibers could compose a nerve trunk, which is wrapped in a myelin sheath^52^. Our data imply that several un-myelinated C-fibers in the masticatory muscles could also be wrapped in myelin sheath, which can be detected by labelling with GFAP. Such organization of C-fibers could not make them a subset of A-fibers, since for A-fiber classification, wrapping in myelin sheath for every neurite is required. Precise organization of C-fibers in these muscles will need to be uncovered using electron microscopy. C-fibers could lose GFAP^+^ sheath during last 30–100 µµ stretch before innervating targets as they do in dura mater^52^.

Primate TG neurons also lack TH labeling in TG. It may indicate that the masticatory muscles do not have C-LTMR. On other hand, extensive network of TH^+^ fibers, which do not co-localize with NFH fibers was detected around blood vessels in MM, TM and LPM. One possible identity for such TH^+^ fibers are sympathetic nerves. These TH^+^ fibers could travel along vessels as a single fiber or organized into bunch/trunk. To clarify this point electron microscopy studies are necessary.

Comparing our results with data from single-cell RNA sequencing of primate DRG neurons imply that masticatory muscle C-fibers are not similar to PEP1 group containing trpV1. PEP2 and PEP3 sensory neuronal groups are peptidergic with no trpV1 expression^44^. However, PEP2 and PEP3 represent A-fibers (A-HTMR groups) and are similar to mouse CGRP-eta^44,59^. Considering that primate masticatory muscles have NFH^+^ peptidergic fibers, which could belong to the A-HTMR group ^53,59^ and be similar to reported PEP2 and PEP3 transcriptomic clusters in primates (Fig. 12). Nevertheless, precise transcriptomic identity of C-fibers and A-HTMR fibers in primate masticatory muscles are not clear.

The remaining A-fibers could be classified as A-LTMR^57^ or CGRP^-^ Aβ-HTMR (Fig. 12). MM, TM and LPM have no expression of calbindin, which is a marker for NF2 cluster in DRG sensory neurons^43^. The cutaneous Aδ-LTMR marker, TrkB, was not present in masticatory muscles (Fig. 12)^46,58^. Parvalbumin is a marker for Aβ-LTMR and was present in few nerves of masticatory muscles. Dominant marker for A-LTMR in MM, TM and LPM was trkC (Fig. 12). Altogether, innervation of the masticatory muscles in marmosets is clearly distinct from cutaneous fibers and primate DRG neurons. Thus, it is unlikely that A-LTMR in masticatory muscles terminate in specialized cell structures, such as Merkel cells, Messner or Pacinian corpuscles as they do in glabrous skin. Moreover, we noted not only similarities, but also differences in nerve types between MM, TM and LPM. These differences can precisely be identified only by detailed single-cell RNA sequencing. Nevertheless, there is a similarity between MM innervation in primates versus mice^20^.

The main conclusions of this study are that (1) the nerves innervating marmoset masticatory muscle are unique compared to cutaneous nerves; (2) innervation nerve subgrouping depend on masticatory muscle types; (3) information available thus far suggests that NHP primate and mouse nerves innervating MM could be quite similar; and (4) highest genetic association of PEP1 and NP2 to human pain states^44^ may not apply for pain conditions in the head and neck area, including TMDM.

## Material and methods

### Animals and ethical approval

The reporting in the manuscript follows the recommendations in the ARRIVE guidelines (PLoS Bio 8(6), e1000412,2010). All animal experiments conformed to IASP and APS's Guiding Principles in the Care and Use of Vertebrate Animals in Research and Training. We also followed guidelines issued by the National Institutes of Health (NIH) and the Society for Neuroscience (SfN) to minimize the number of animals used and their suffering. All animal experiments conformed to protocols approved by the University Texas Health Science Center at San Antonio (UTHSCSA) and Texas Biomedical Research Institute (TBRI, San Antonio, TX) Institutional Animal Care and Use Committee (IACUC). IACUC protocol title is “Plasticity of Lymphotoxin-beta signaling and Orofacial pain in non-human primates”, and numbers are 20200021AR from UTHSCSA and 1821 CJ 0 from TBRI.

For our studies, we collected tissues from 4, 5 and 11 years-old adult marmosets. MM was collected from right and left sides of 4, 5, 5 and 11 years-old NHPs, while right and left TM and LPM was isolated only from 5, 5 and 11 years-old marmosets. Tissues were collected within 2 h post NPH euthanasia. Animals were housed at the UTHSCSA or TBRI. Samples for this study were collected opportunistically, including “Tissue Share program in UTHSCSA and TBRI, from animals that were euthanized at IACUC or TBRI approved endpoints on their respective studies. Marmosets used in this study did not have either injury affecting the head and neck area, or systemic infections.

### Tissue collection and processing

Animals were euthanized by the veterinarians in UTHSCSA and TBRI at defined end points that were determined for each animal. At the point of determined death, superficial and deep heads of MM, TM, superior and inferior heads of LPM with attached TMJ ligament, TG, dorsal root ganglia (DRG), hindpaw skin, dura mater, and tongue were dissected and placed in 4% paraformaldehyde (PFA) as previously described^34^. Tissues were fixed in 4% PFA for 3–4 h, washed in 3 × 15 min in 0.1 M Phosphate Buffer (PB), equilibrated in 10% sucrose in PB at 4 °C overnight and cryo-protected and stored in 30% sucrose in PB at − 20 °C. Tissues were embedded in Neg 50 (Richard Allan Scientific, Kalamazoo, MI); and were cryo-sectioned with the following thickness: TG 20 µm and MM, TM and LPM 30 µm.

### Immunohistochemistry (IHC)

Immunostaining was performed as described previously^71,72^. Briefly, sections were blocked in 4% normal donkey serum (Sigma, St. Louis, MO), 2% bovine gamma-globulin (Sigma-Aldrich, St. Louis, MO) and 0.3% Triton X-100 (Fisher Scientific) in 0.1 M PBS for 90 min at RT. Next, tissue sections were incubated for 24–36 h at RT with primary antibodies. Sections were washed with PBS from unbound primary antibodies, blocked, and incubated for 90 min at RT with appropriate fluorophore conjugated secondary antibodies (Jackson Immuno-Research, West Grove, PA, USA). Finally, tissue sections were washed for 3 × 5 min with 0.1 M PBS and 2 × 5 min in diH_2_O, air-dried, and covered with Vectashield Antifade Mounting Medium (Vectorlabs, Burlingame, CA, USA). In this study, the following previously characterized primary antibodies were used: anti-PGP9.5 rabbit polyclonal (Millipore-Sigma; Burlington, NJ, catalog AB1761-I; 1:300)^73^; anti-Neurofilament H (NF-H) chicken polyclonal (BioLegend; San Diego, CA; catalog 822,601; 1:1000)^74^; anti-CGRP guinea pig polyclonal (Synaptic Systems; Goettingen, Germany; catalog 414 004; 1:200)^75^; anti-TRPV1 rabbit polyclonal (Novus Biologicals; Centennial, CO; catalog NBP1-71774SS; 1:200)^76^; anti-mrgprD rabbit polyclonal (Alamone Lab; catalog ASR-031; 1:200)^20,77^; anti-CHRNA3 rabbit polyclonal (Bioss; catalog BS-6455R; 1:200)^78^; anti-tyrosine hydroxylase (TH) chicken polyclonal (Neuromics; Bloomington, MN; catalog CH23006; 1:300)^79^; anti-smooth muscle actin (α-SMA) Cy3-conjugated rabbit polyclonal antibody (Millipore-Sigma; catalog C6198; 1:500); anti-trkC rabbit polyclonal (Aviva Systems Biology, San Diego, CA; catalog ARP51318_P050; 1:200); anti-trkB goat polyclonal (R&D systems; AF1494; 1:200)^80^; anti-parvalbumin rabbit polyclonal (Novus Biologicals; catalogue NB120-11427SS; 1:200)^81^; anti-calbindin D28k rabbit polyclonal (Synaptic Systems; catalogue 214 011; 1:200)^82^.

### Counting of nerve fibers and neurons

Images were acquired using a Keyence BZ-X810 all-in-one microscope (Itasca, IL, USA) in the “sectioning” mode. Images were acquired with a 2 × , 10 × or 20 × objective. Control IHC was performed on tissue sections processed as described but either lacking primary antibodies or lacking primary and secondary antibodies. Settings were determined in such way that no-primary antibodies and both no-primary and no-secondary antibody controls did not show any positive signal. Then, images were taken using these fixed acquisition parameters across all groups. For cell and fiber counting, Z-stack IHC images with × 10 or × 20 objectives were obtained from 3 to 5 independent tissue sections from 2–3 primates/isolations. Counting was normalized to field of view of × 10 objective, which is fourfold large than for × 20 objective. Neuronal fibers were counted manually as described using Image J software^34^ to obtain approximate estimation of the numbers of peripheral nerve types innervating muscle tissues. Positively labeled nerve fibers were distinguished from labeling of non-neuronal cells or artefacts by clear visual identification of fibers with at least 20 µµ length (Fig. 3, middle panel). Moreover, bundles of fibers grouped together and with at least 20 µµ length that couldn’t be visually separated were also counted as 1. The nerve fiber for counting were identified according to morphology; thus, non-specific labeling or labeling non-neuronal cells were not counted. Mean values from these counting from 3 to 5 sections generated from a NPH represented data for a biological replicate. Thus, n = 3 means represent 3 NHP as the biological replicates.

### Statistical analyses

GraphPad Prism 8.0 (GraphPad, La Jolla, CA) was used for statistical analysis. Data are presented as mean ± standard error of mean (SEM). Differences between groups were assessed by chi-square analysis with Fisher’s exact test, unpaired t-test, or 1-way ANOVA with Bonferroni’s post-hoc test. A difference is accepted as statistically significant when *p* < 0.05. Interaction F ratios, and the associated *p* values are reported.

### Ethical approval and informed consent

The reporting in the manuscript follows the recommendations in the ARRIVE guidelines (PLoS Bio 8(6), e1000412,2010). All animal experiments conformed to IASP and APS's Guiding Principles in the Care and Use of Vertebrate Animals in Research and Training. We also followed guidelines issued by the National Institutes of Health (NIH) and the Society for Neuroscience (SfN) to minimize the number of animals used and their suffering. All animal experiments conformed to protocols approved by the University Texas Health Science Center at San Antonio (UTHSCSA) and Texas Biomedical Research Institute (TBRI, San Antonio, TX) Institutional Animal Care and Use Committee (IACUC). IACUC protocol title is “Plasticity of Lymphotoxin-beta signaling and Orofacial pain in non-human primates”, and numbers are 20200021AR from UTHSCSA and 1821 CJ 0 from TBRI.

### Supplementary Information

Supplementary Information.
