# Supplementary material for: Sensory innervation of masseter, temporal and lateral pterygoid muscles in common marmosets
Source: Sci Rep. 2023 Dec 27;13:23062. doi: 10.1038/s41598-023-49882-9 (PMC10754842; doi:10.1038/s41598-023-49882-9)
Supplement: Supplementary file 1 — Supplementary Information. [file 41598_2023_49882_MOESM1_ESM.pdf]

Legends to Supplementary Figures:

Supplementary Figure 1. ***Location of pgp9.5 and NFH-positive fibers in MM, TM and LPM of adult marmosets.***

Left column shows schematic for sensory nerves in marked/specified masticatory muscles. *Middle column* shows expression of pgp9.5 and NFH-positive fibers in MM, TM and LPM. Right column shows expression of NFH-positive fibers in MM, TM and LPM. Pictures from MM, TM and LPM as well as antibodies used and corresponding colors are indicated. Scales are presented in each microphotograph.

Supplementary Figure 2. ***Positive control experiments with antibodies for nociceptors, "small" sensory neurons, and glia***.

Top-row panels show expression of CGRP (peptidergic neurons) and trpV1 in TG of adult male marmoset. *Middle row panels* show expression of GFAP (pan glial marker), mrgprD (a marker for non-peptidergic neurons) and tyrosine hydroxylase (TH; a marker for C-fiber low threshold mechanoreceptor (C-LTMR)) in in TG of adult male marmoset. *Bottom row panel* exhibits location of CHRNA3 (a marker of "silent" nociceptors in DRG) in tongue of adult male marmosets. Yellow arrows mark CHRNA3$^+$ fibers in a marmoset tongue section labeled with CHRNA3 and NFH. Antibodies used and corresponding colors are indicated. Scales are presented in microphotographs from top two rows.

Supplementary Figure 3. ***Distribution of peptidergic fibers in MM, TM and LPM of adult marmosets***. Representative micro-photographs show CGRP-positive peptidergic fiber distributions relative to NFH$^+$ fibers in MM, TM, and LPM of adult male marmosets. Yellow arrows indicate CGRP$^+$/NFH$^+$ fibers, cyan arrows show CGRP$^+$/NFH$^-$ fibers, and blue arrows mark CGRP$^-$

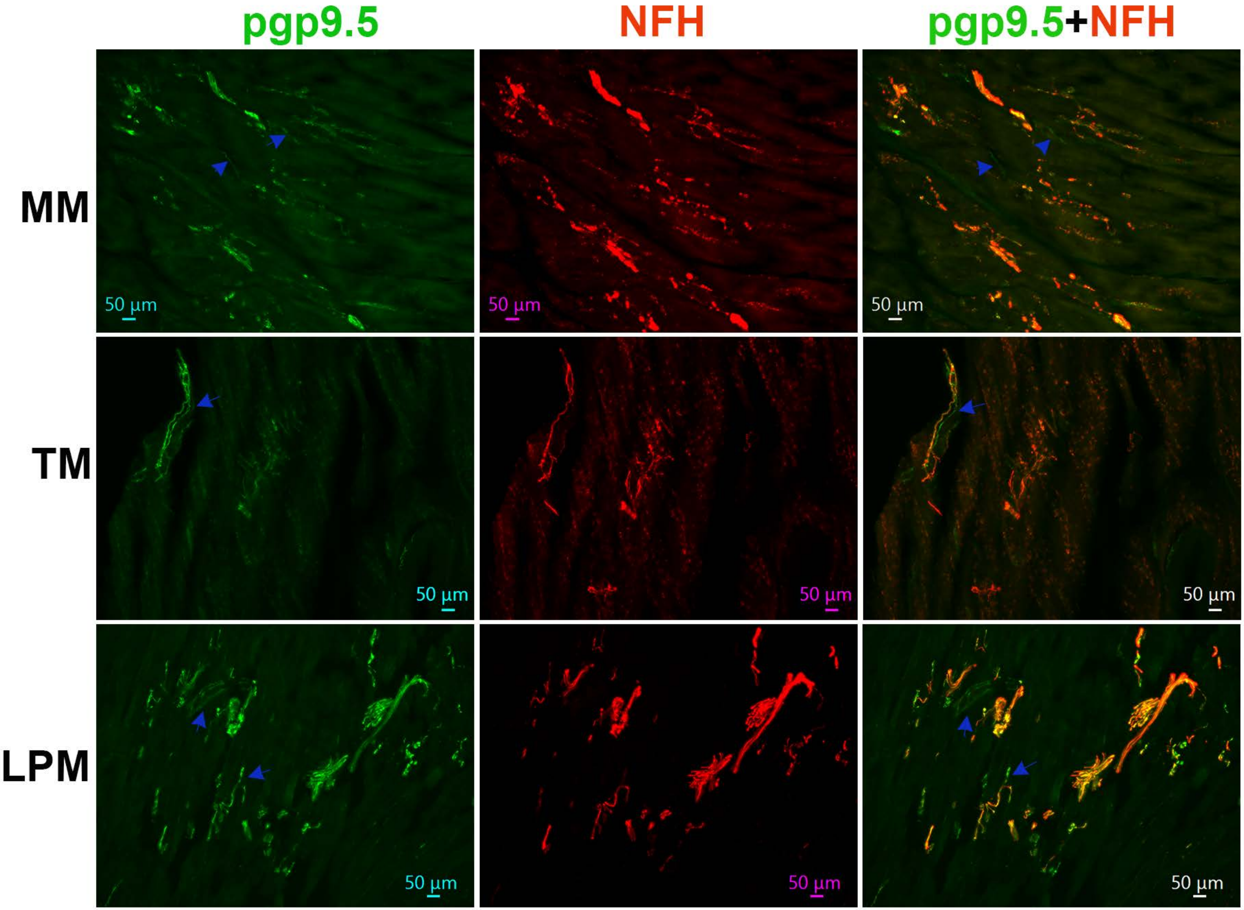

Suppl Fig 1

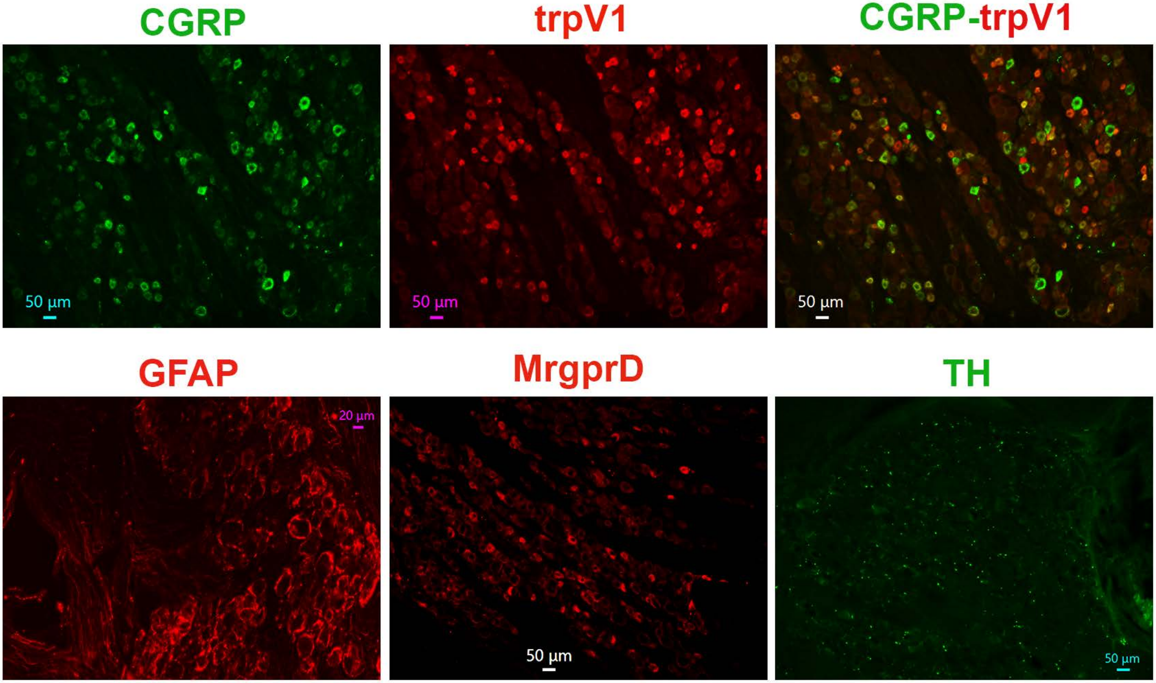
CGRP
trpV1
CGRP-trpV1
50 µm
50 µm
50 µm
GFAP
MrgprD
TH
20 µm
50 µm
50 µm

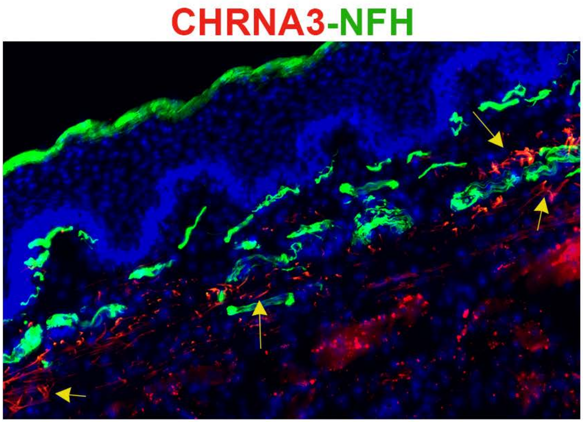
CHRNA3-NFH

Suppl Fig 2

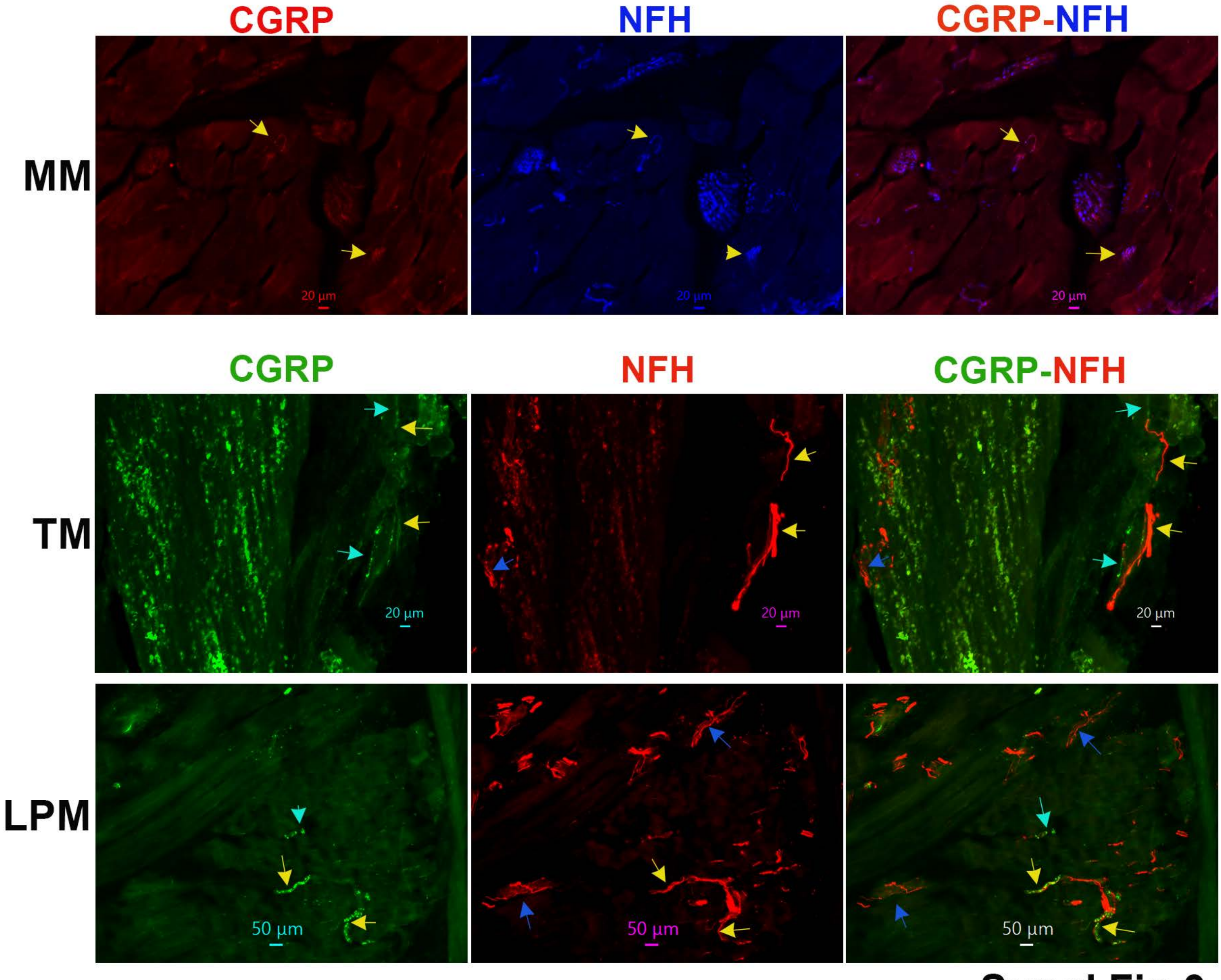
CGRP
NFH
CGRP-NFH
MM
20 μm
20 μm
20 μm
CGRP
NFH
CGRP-NFH
TM
20 μm
20 μm
20 μm
LPM
50 μm
50 μm
50 μm

Suppl Fig 3

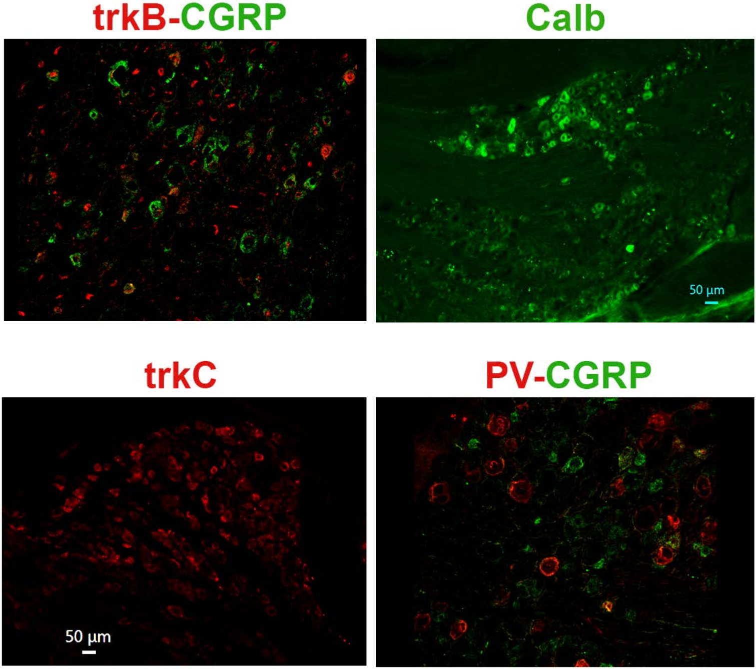

Suppl Fig 4

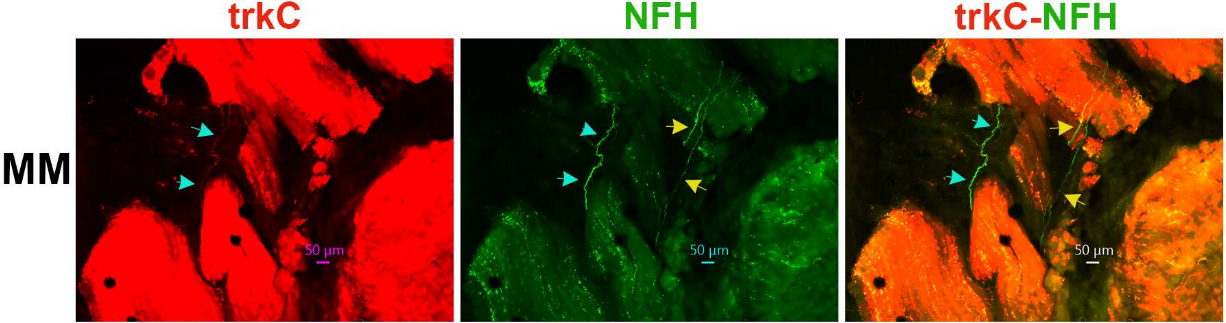

Suppl Fig 5
